# A Comparison of Spectroscopy and Imaging Techniques Utilizing Spectrally Resolved Diffusely Reflected Light for Intraoperative Margin Assessment in Breast-Conserving Surgery: A Systematic Review and Meta-Analysis

**DOI:** 10.3390/cancers15112884

**Published:** 2023-05-23

**Authors:** Dhurka Shanthakumar, Maria Leiloglou, Colm Kelliher, Ara Darzi, Daniel S. Elson, Daniel R. Leff

**Affiliations:** 1 Department of Surgery and Cancer, Imperial College London, London W12 0HS, UK; 2 The Hamlyn Centre, Imperial College London, London SW7 2AZ, UK

**Keywords:** tissue optics, breast cancer, breast-conserving surgery, hyperspectral imaging, diffuse reflectance spectroscopy

## Abstract

**Simple Summary:**

Breast-conserving surgery (BCS) is an oncological procedure that allows for the excision of breast cancer with a clear margin of healthy tissue whilst optimising the cosmetic appearance. However, BCS is associated with up to a 19% re-excision rate due to incomplete excision (“positive margins”) in the United Kingdom. Optical spectroscopy and the optical imaging of BCS specimens could be a potential intraoperative margin assessment tool to help reduce re-excision rates. Hyperspectral sensing is based on the premise that light illuminating biological tissues undergoes several processes that reflect the composition of tissue, thus helping to differentiate between normal and malignant tissues. This review assesses the current literature on the use of hyperspectral sensing in breast cancer. We divide the techniques into either point-based (spectroscopy) or whole field-of-view (imaging) methods. A comparison is made of the effectiveness of these modalities in discriminating between normal and malignant tissue, and we reflect on the usability of these modalities in the intraoperative setting.

**Abstract:**

Up to 19% of patients require re-excision surgery due to positive margins in breast-conserving surgery (BCS). Intraoperative margin assessment tools (IMAs) that incorporate tissue optical measurements could help reduce re-excision rates. This review focuses on methods that use and assess spectrally resolved diffusely reflected light for breast cancer detection in the intraoperative setting. Following PROSPERO registration (CRD42022356216), an electronic search was performed. The modalities searched for were diffuse reflectance spectroscopy (DRS), multispectral imaging (MSI), hyperspectral imaging (HSI), and spatial frequency domain imaging (SFDI). The inclusion criteria encompassed studies of human in vivo or ex vivo breast tissues, which presented data on accuracy. The exclusion criteria were contrast use, frozen samples, and other imaging adjuncts. 19 studies were selected following PRISMA guidelines. Studies were divided into point-based (spectroscopy) or whole field-of-view (imaging) techniques. A fixed-or random-effects model analysis generated pooled sensitivity/specificity for the different modalities, following heterogeneity calculations using the Q statistic. Overall, imaging-based techniques had better pooled sensitivity/specificity (0.90 (CI 0.76–1.03)/0.92 (CI 0.78–1.06)) compared with probe-based techniques (0.84 (CI 0.78–0.89)/0.85 (CI 0.79–0.91)). The use of spectrally resolved diffusely reflected light is a rapid, non-contact technique that confers accuracy in discriminating between normal and malignant breast tissue, and it constitutes a potential IMA tool.

## 1. Introduction

Breast cancer is the most commonly diagnosed cancer internationally, and the most common cancer among females [1]. The preferred management method for early-stage breast cancer is breast-conserving surgery (BCS) [2]. In BCS, the aim is to excise the cancer with adequate margins while preserving cosmetic outcomes. Intraoperatively, the surgeon locates the cancer through a combination of pre-operative localization techniques (e.g., seed, wire, etc.), and palpation. However, surgeons do not know precisely where the cancer ends and normal tissue begins and risk cutting too close to the cancer perimeter, the so-called “positive margin”. Achieving adequate tumour clearance with a rim of normal tissue, or a “clear margin”, is crucial, as patients with positive margins have a higher risk of local recurrence and therefore require re-excision surgery [3,4].

Unfortunately, a substantial number of women undergo re-operative intervention for positive margins [5,6]. For example, a recent national ‘Getting It Right The First Time’ initiative recorded a UK national average re-operation rate of 19% [7]. Re-operative interventions have a significant psychosocial impact [8,9]. There are delays in adjuvant treatment [10], which consequentially affect quality-of-life outcomes and perceptions of cancer care [9], as well as placing a financial burden on the tax payer [11].

The GIRFT report stated that strategies need to be sought to reduce re-excision rates, and intraoperative assessment tools are one possible strategy [7]. Optical technologies offer the opportunity to extract structural and morphological information from biological tissues using light–tissue interactions. Harnessing this knowledge in an intraoperative tool may allow us to accurately distinguish between malignant and benign tissue. The key clinical advantages of optical technologies are their non-ionizing and non-invasive properties, with the potential to provide the surgeon with near real-time feedback [12].

Understanding light–tissue interactions is crucial to creating a useful intraoperative tool. Light delivered to biological tissues undergoes several processes [13] including reflection and refraction at the surface, and scattering from tissue structures and cellular components. In breast tissue, scattering is sensitive to breast density (reflective of fibroglandular content [14]) and collagen; increased collagen deposition is involved in tumour progression [15]. Furthermore, light may be absorbed by molecules called “chromophores”, which include water, lipids, and haemoglobin [16]. 

A further interaction is fluorescence, whereby light energy is absorbed by fluorophores in the tissue (either naturally occurring or molecular probes) and then re-emitted at a longer ‘red-shifted’ wavelength. In breast tissue, inherent fluorescent molecules include nicotinamide adenine dinucleotide and hydrogen (NADH), flavin adenine dinucleotide (FAD), collagen, elastin and tryptophan, and lipo-pigments [15]. Fluorescence in the visible and near-infrared spectral ranges is typically a weaker interaction than scattering or absorption, although it becomes more significant for shorter wavelength (ultraviolet) illuminations.

Although an array of optical technologies is being investigated worldwide, we have focused this review on literature in which the use of diffusely reflected light has been assessed in relation to breast cancer detection in the intra-operative setting. We concentrated on evaluating only papers that exploit endogenous breast tissue properties, rather than relying on exogenous agents. To our knowledge, there has been no recent review appraising the effectiveness of technology that uses spectrally resolved diffusely reflected light in the intraoperative setting. Such work enables us to evaluate whether the utilization of diffusely reflected light has potential as an intraoperative margin assessment tool, and what further research is required to progress the field.

**Figure 1:**
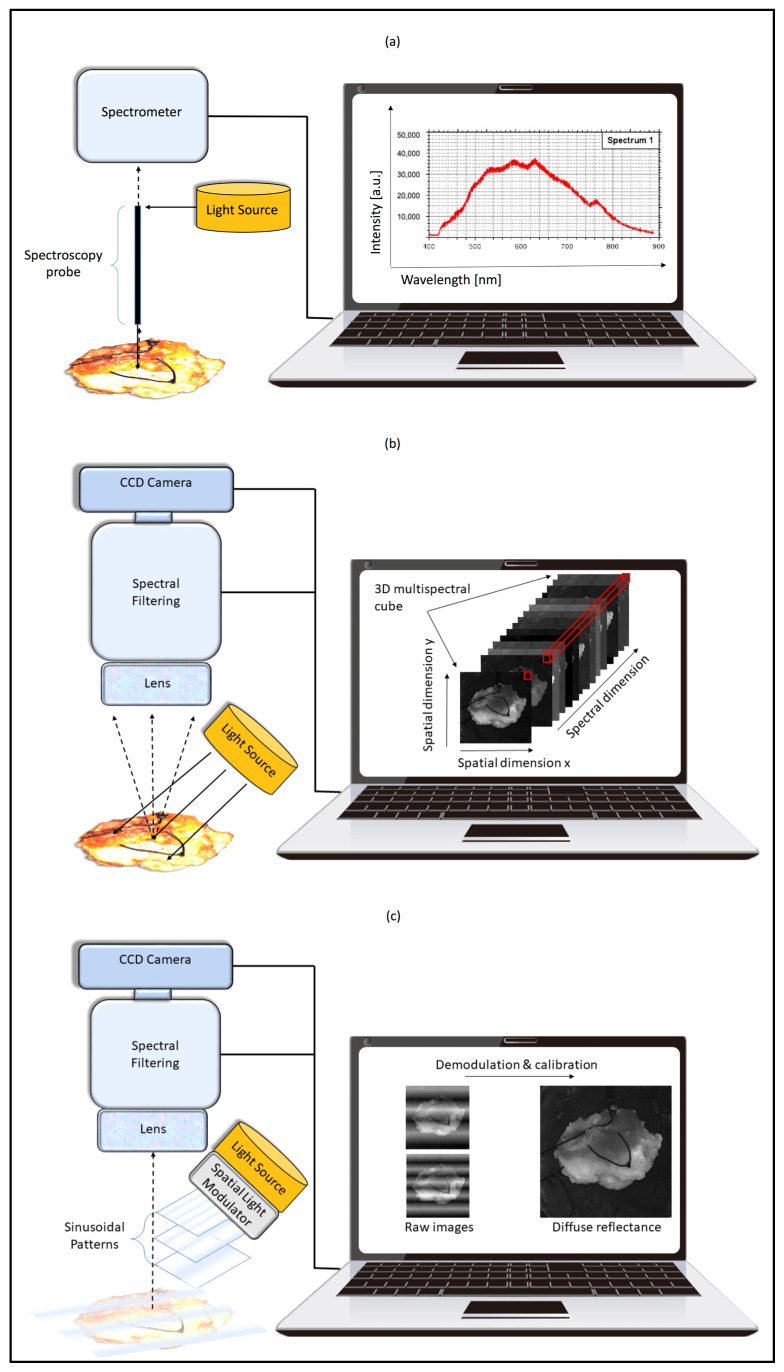
Schematic of the three imaging categories: (**a**) diffuse reflectance spectroscopy, (**b**) multispectral/hyperspectral imaging, (**c**) spatial frequency domain imaging. (**a**) With diffuse reflectance spectroscopy, a probe is applied to a tissue sample. One of the many fibres of the probe transmits light to the tissue. Diffusely reflected light from the tissue is collected by different fibres within the probe and is measured by a spectrometer. A spectrometer takes in the light through a narrow slit, which is then reflected onto a concave mirror. The collimating light beam that is produced is directed onto a diffraction grating. The grating disperses the spectral components of light at varying angles, and it is then focused by a second concave mirror and imaged onto a detector. The detector measures the amount of light absorbed at each wavelength, and then digitalizes the signal as a proportional electrical signal, which is displayed via a computer. (**b**) Multispectral or hyperspectral imaging systems use a lens that captures light reflected from a tissue sample, which has been illuminated with light from an external source. Light enters the spectral filtering component, which selectively transmits light according to its wavelength. Similarly to spectroscopy, this component disperses each wavelength of light to focus onto a charged couple device (CCD). A CCD is a sensor that breaks the two-dimensional image elements into pixels. Each pixel (depicted by a red square) represents a spectral band or part of the electromagnetic spectrum. A three-dimensional datacube is generated, which comprises of a set of two-dimensional images of a sample and records the spectral information of each pixel in the image. Multispectral imaging provides discrete and discontinuous portions of the spectral range, whereas hyperspectral imaging uses a larger number of contiguous bands. (**c**) With spatial frequency domain imaging systems, a projector illuminates the target tissue area with a two-dimensional light pattern composed of various frequency modulations of a sinusoidal wave. The reflectance pattern of this sinusoidal patten is captured by the camera lens. The CCD component picks up the emitted diffuse light, which then undergoes demodulation by a computer to extract diffuse reflectance. Demodulation is a process that calculates amplitude modulation for every pixel of the image. Optical properties per pixel are extracted using light propagation models.

The methodologies resulting from this review can be categorized into three areas, represented in Figure 1, based on the instrumentation and signal processing they entail:

(a) Diffuse reflectance spectroscopy

Diffuse reflectance spectroscopy (DRS) measures the intensity of diffusely reflected light as a function of wavelength [17,18]. For DRS measurements, a fibre optic probe is in contact with the tissue. This probe contains several fibres, one of which is connected to a broadband light source and transmits light to the tissue being studied (Figure 1a). Light is then diffusely reflected (sensitive to absorption and scattering) in the tissue and collected by different fibres within the probe for measurement by a spectrometer. Tissue morphology affects the amount of absorption and scattering of light, which can then be inferred from changes in the DRS spectrum.

Various methods allow for the spectral analysis of diffuse reflectance, enabling the extraction of useful information about the optical properties of biological tissues. This is described as diffuse reflectance modelling. Most methods used to model diffuse reflectance from biological tissues involve approximations of the radiative transport equation (such as diffusion theory), which incorporate potential inaccuracies and deviations into the final model [19,20]. Monte Carlo simulations are commonly used for spectral analysis. This statistical method relies on calculating the propagation of a large number of photons. Therefore, data processing requires long computational times [21].

This review does not evaluate literature where exogenous agents are used to enhance fluorescence. However, the use of fluorescence spectroscopy is evaluated with ‘intrinsic fluorescence spectroscopy (IFS)’, as it is used with DRS in several studies. Here, the acquired fluorescence signal is leveraged to probe the presence of inherent fluorophores [22]. Fluorescence spectroscopy, as a separate entity, is not evaluated as it lies outside the scope of this review.

(b) Multispectral/Hyperspectral Imaging

Spectral imaging is a technology that combines conventional imaging with spectroscopy (Figure 1b) to obtain the spatial and spectral information from an object [23]. Traditional optical imaging techniques, such as red–green–blue cameras, only use three visible bands of light and have limited identification capabilities. Spectral imaging uses significantly more bands, helping to identify the alterations in tissue caused by tumour progression. Spectral imaging can be divided into either multispectral (MSI) or hyperspectral (HSI) categories, depending on the number of the acquired spectral bands, or on the spectral resolution [23]. 

A spectral band represents a segment of the electromagnetic (EM) spectrum. The human eye only perceives light in the visible range (400–700 nm), which is a very small portion of the EM spectrum, but MSI/HSI systems frequency acquire bands from the ultraviolet through to the near-infrared (NIR) spectral ranges. MSI/HSI creates a three-dimensional dataset called a ‘hypercube’ which has both spatial and spectral coordinates [24]. The benefit of the NIR range is that it augments the vision of the human eye and has the ability to penetrate tissue from several millimetres (mm) to centimetres (cm) due to reduced scattering and absorption [25]. MSI/HSI allows for a greater area of tissue to be imaged in real time compared to probe-based DRS.

(c) Spatial Frequency Domain Imaging

Spatial frequency domain imaging (SFDI) has the ability to separate the effects of the absorption and scattering of tissue. SFDI projects a two-dimensional light pattern onto a sample, which consists of sinusoidal stripes of varying spatial frequencies (i.e., stripes per mm) and a digital camera captures the reflectance image (Figure 1c). Due to the absorption and scattering within the medium, the visibility of the projected pattern decreases, resulting in a measurable change in the modulation depth. The demodulation is calculated for every pixel of an image for several spatial frequencies, from which a light propagation model can be used to extract the optical properties [26].

## 2. Materials and Methods

### 2.1. Literature Search Methodology

A literature review was conducted as per the guidelines for the ‘Preferred Reporting Items for Systematic Reviews and Meta-analyses’ (PRISMA). An electronic search of the Medline, Embase, and Scopus databases were conducted. Relevant studies from July 1985 to December 2021 were identified. Suitable search terms defining both ‘breast cancer’ and ‘breast surgery’ were identified. These were then combined using the Boolean operator ‘AND’ for search terms identifying the optical technologies being investigated in this review. The aim was to identify the use of the discussed modalities in the intraoperative setting, rather than the pathology setting. A combination of ‘Medical Subject Headings’ (MeSH) and free-text words were identified to capture the various aspects of the research question. Relevant papers were imported into Covidence software (Veritas Health Innovation, Melbourne, Australia), where duplicate papers were removed. The full search strategy is available in the Supplementary Materials. The review and meta-analysis were registered with PROSPERO (CRD 42022356216).

### 2.2. Selection Criteria

**Figure 2:**
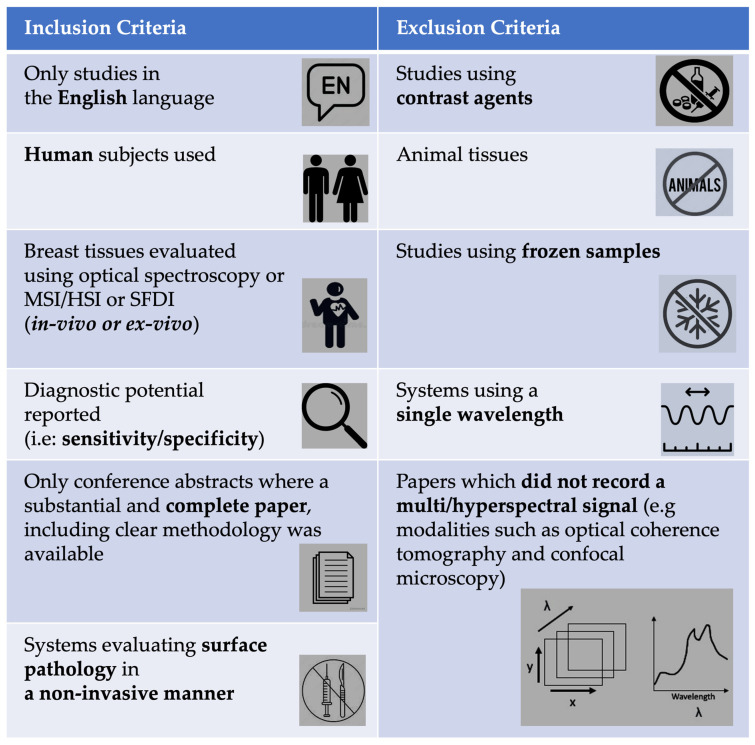
Inclusion and exclusion criteria used to screen abstracts.

Title and abstract screening were conducted according to pre-defined inclusion and exclusion criteria (Figure 2). 

### 2.3. Data Collection

Independent assessment by two investigators (D.S. and C.K.) was conducted using Covidence software. Any conflicts were discussed and resolved with explanations of ‘yes’, ‘no’, or ‘uncertain’. All ‘uncertain’ cases underwent full-text screening and were discussed with M.L. and D.E. A pre-defined Excel spreadsheet was used to collate the required information. Data extraction included the sample size, the wavelength of light, the type of modality used and its type (i.e., probe geometry/imaging set-up), the data acquisition time, the tissue area sampled, the tissue histology types, and the diagnostic potential to detect cancer. 

### 2.4. Meta-Analysis

**Figure 3:**
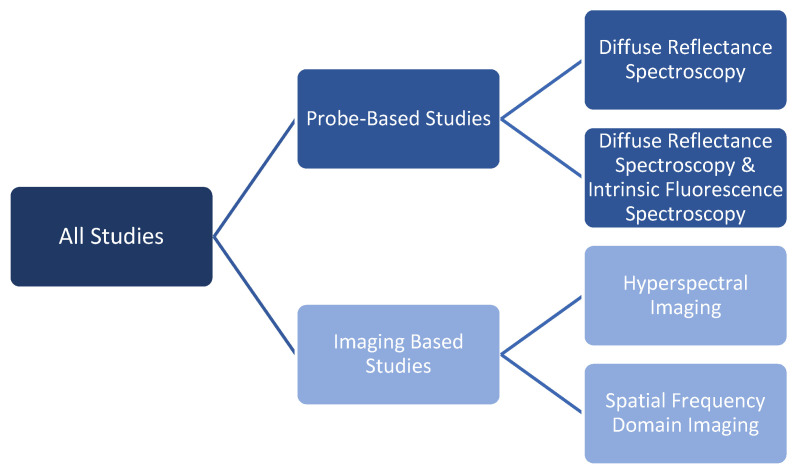
Studies were divided into probe-based or imaging-based studies. Within these groups, further subdivisions were made.

Before the meta-analysis, the studies were divided into two main categories: probe-based studies or imaging-based studies. Within these categories, further subdivisions were made, as depicted in Figure 3. Specifically, probe-based studies were divided into diffuse reflectance spectroscopy (DRS) and diffuse reflectance combined with intrinsic fluorescence spectroscopy (DRS-IFS). Imaging-based studies were divided into hyperspectral imaging (HSI) and spatial frequency domain imaging (SFDI). 

Heterogeneity between the studies for each main group and for the subsequent group subdivisions was calculated using the Q statistic. The null hypothesis was that the sensitivity (or the specificity) was the same in all of the studies and that any between-study variations in sensitivity (or specificity) existed only due to sampling errors. After extraction of the Q statistic, the corresponding *p*-value was calculated with the use of the cumulative chi-squared distribution and with N-1 degrees of freedom, where N was the number of studies in each group. Alongside the *p*-value of the Q statistic, the posterior probability for heterogeneity Pr(Het|Q) was used as a between-study heterogeneity indicator [27]. This probability was calculated according to Bayes’ theorem, taking into consideration the power of the Q test, the significance level used (a = 0.05), and a prior probability of heterogeneity [28]. This prior probability reflected the observed between-study heterogeneity, including the tissue samples and hardware equipment used and data acquisition/processing and ground truth extraction methods. In the cases where the null hypothesis could not be rejected, the posterior probability Pr(Het|Q) was close to the prior probability for heterogeneity for a Q test of low power or far from the prior probability for a Q test of high power. Both fixed- and random-effects model analyses were used to extract pooled sensitivity/specificity. Finally, the pooled sensitivity/specificity values for the probe-based studies were compared with those of the image-based studies with the help of the Q-statistic and chi-squared distribution. The same methodology was also used to compare the pooled results between the subdivisions (DRS, DRS-IFS, HSI, or SFDI) within the probe-based and image-based studies.

## 3. Results

**Figure 4:**
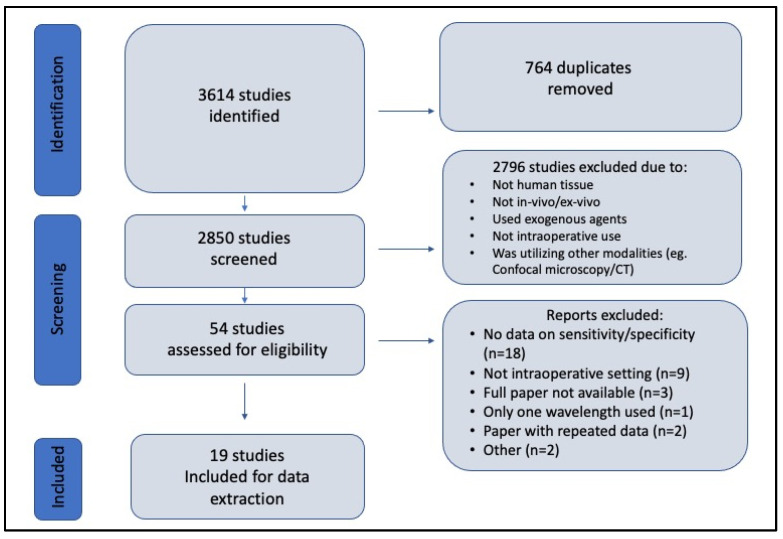
PRISMA flowchart showing the study selection process.

Overall, 3613 studies were identified from the literature search, of which 19 met the inclusion criteria (Figure 4).

### 3.1. Categorization of Selected Studies

#### 3.1.1. Tissue Optic Modality Type

Of the nineteen studies eligible for this study (Table 1), thirteen used probe-based systems, and six used image-based modalities. In terms of image-based modalities, the selected studies used the phrases ‘hyperspectral imaging’ and ‘spatial frequency domain imaging’. Although ‘multispectral imaging’ was searched for, no studies using this phrase were found for intraoperative applications, although some of the hyperspectral studies may be termed multispectral depending on the preferred definitions of these terms. All studies used ex vivo samples, and no in vivo work was identified. Most studies used the visible wavelength range [14,29,30,31,32,33,34,35,36,37,38]; however, six included the use of the near-infrared range [39,40,41,42,43,44,45].

#### 3.1.2. Probe-Based Systems vs. Imaging-Based Systems

An ideal IMA tool provides the surgeon with rapid visualization of the region of interest. Of the nineteen studies, only six used whole field-of-view imaging modalities (Table 1). The rest used probe-based technology to identify malignancy in breast tissue. The set-up of the probe systems was variable (Table 2). Probe geometry varied in terms of being either single or multichannel, and the inter-fibre distance varied.

Although the search term “multispectral imaging” was used, no papers were identified using this term exactly; therefore, the imaging-based studies were divided into HSI and SFDI, as per Figure 2. The advantage of these technologies is that a larger area of tissue can be examined more rapidly. Some studies quantified the maximum areas their technology can visualize (Table 3). The areas described (mean (StD) = x(y)) are significantly larger than those evaluated by any probe-based system.

### 3.2. Tissue Heterogeneity among Studies

Specimen parameters that must be taken into consideration include the patient demographics, such as age, body mass index, and menopausal status, as well as breast density. Pre-menopausal women have more fibroglandular tissue and denser breast tissue. Very few of the evaluated studies provide a clear description of patient demographics.

Breast cancer is heterogenous, with varying molecular and histological subtypes and immunophenotypes [47,48]. While ductal cancer is the most common (85%), many patients present with lobular breast cancer (10–15%) and rarer subtypes. Ten of the reviewed studies evaluated different histological subtypes (Table 4). Receptor status, such as oestrogen (ER), progesterone (PR), and HER2, dictate the modern medical management of breast cancer, including neoadjuvant chemotherapy for triple-negative and ER-/HER2+ breast cancers. Only two studies included patients who had received neoadjuvant chemotherapy.

There are several well-documented predictors for positive margins, namely, the presence of ductal carcinoma in situ (DCIS), the lobular tumour type, and a larger tumour size [49,50,51]. Therefore, any future IMA tool must be able to identify different histological subtypes. Nevertheless, as Table 4 illustrates, patients with either lobular cancer or DCIS were not represented in six studies.

### 3.3. Diagnostic Abilities of Different Tissue Optic Techniques

Future intraoperative margin assessment tools using light–tissue interactions must have the diagnostic ability to distinguish between normal and malignant tissues. In this review, we evaluated the sensitivity/specificity of each method (Table 5). The pooled sensitivity/specificity of each modality is shown in Section 3.4. Diagnostic accuracy was reported in five studies [33,39,41,44,45]; however, without the provision of true positive/negative rates for all studies, we were unable to calculate pooled accuracy rates.

### 3.4. Meta-Analysis

#### 3.4.1. Heterogeneity Results

The Q-statistics, which indicate the between-study heterogeneity in sensitivity (or specificity) for each main group and subsequent group subdivisions, are presented below in Table 6 and Table 7, respectively. In both Tables, it is evident that the *p*-values are greater than 0.05, and therefore the null hypothesis cannot be rejected. This suggests that a fixed-effect model analysis would be appropriate for extracting the pooled sensitivity/specificity. However, the pooled results from the random-effects model are also presented in the following section. This is because the power of the Q test is very low (third column), and the posterior probability for between-study heterogeneity (fourth column) strongly depends on the prior probability for heterogeneity. Table 3 and Table 4 clearly demonstrate that between-study variation in demographics, imaging equipment, and geometry does exist. Therefore, a high prior probability of heterogeneity would be appropriate. However, here, we used a conservative prior probability of Pr(Het) = 0.5 to demonstrate the strong dependence of the posterior (Pr(Het|Q)) on the prior probability (Pr(Het)).

#### 3.4.2. Pooled Sensitivity/Specificity Results

Table 8 shows the pooled sensitivity and specificity with the corresponding lower ($S_{pooled}-{S.E.}_{S_{pooled}})$ and higher ${(S}_{pooled}+{S.E.}_{S_{pooled}})$ limits for probe-based approaches compared to image-based approaches. Similarly, the pooled sensitivity/specificity was calculated for the modality subdivisions (presented in Table S2, Supplementary Materials). The number outside the parenthesis is the result from the fixed-effects model analysis, whereas the number in the parenthesis is the result from the random-effects model analysis. Results indicated with an asterisk (*) are categories where the Q-statistic was very small (Q < 1). This means that the study heterogeneity within these categories was very low. The resulting tau-squared value (which represents the between-study variance) was negative and was set to zero. This resulted in $w_{i}^{*}=w_{i}$, and the results of the random-effects model match those of the fixed-effects model.

**Figure 5:**
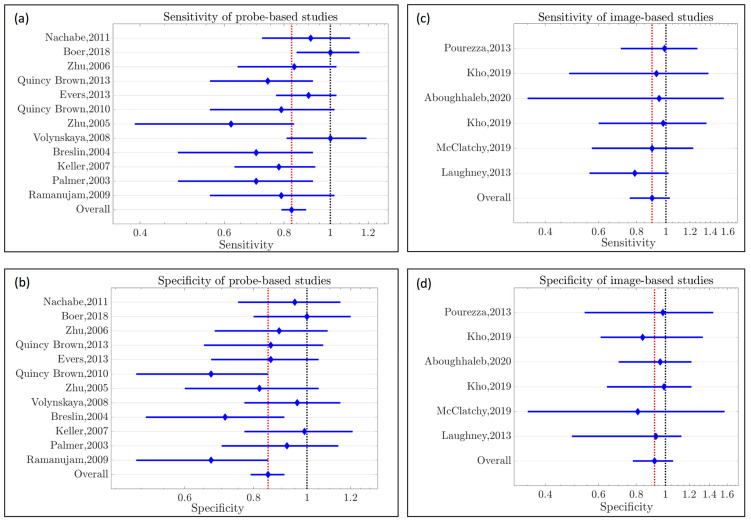
(**a**) Forest plot of the probe-based studies [19,20,21,22,23,24,25,26]; a fixed-model analysis provided a pooled sensitivity of 0.84, with a lower limit of 0.78 and an upper limit of 0.89. (**b**) Forest plot of the probe-based studies [19,20,21,22,23,24,25,26]; a fixed-model analysis provided a pooled specificity of 0.85, with a lower limit of 0.79 and an upper limit of 0.91. (**c**) Forest plot of the image-based studies; a fixed-model analysis provided a pooled sensitivity of 0.90, with a lower limit of 0.76 and an upper limit of 1.03. (**d**) Forest plot of the image-based studies [27,28,29,30,31,32,33,34,35,36,37,38,39,40]; a fixed-model analysis provided a pooled specificity of 0.92, with a lower limit of 0.78 and an upper limit of 1.06 [27,28,29,30,31,32,33,34,35,36,37,38,39,40].

**Figure A1:**
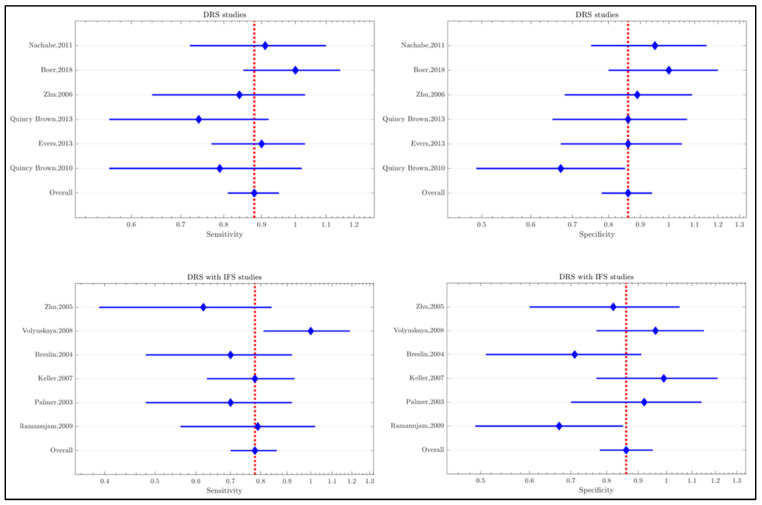
Forest plots for pooled sensitivity and specificity applied to the subdivisions within probe based studies [14,29,30,31,32,33,34,35,36,39,40,41,46]. Forest plots for pooled sensitivity (**left**) and specificity (**right**) where a fixed-model analysis was applied to the DRS studies [14,29,30,39,40,41] only (**top**) and to the DRS with IFS studies [31,32,33,34,35,46] only (**bottom**).

**Figure A2:**
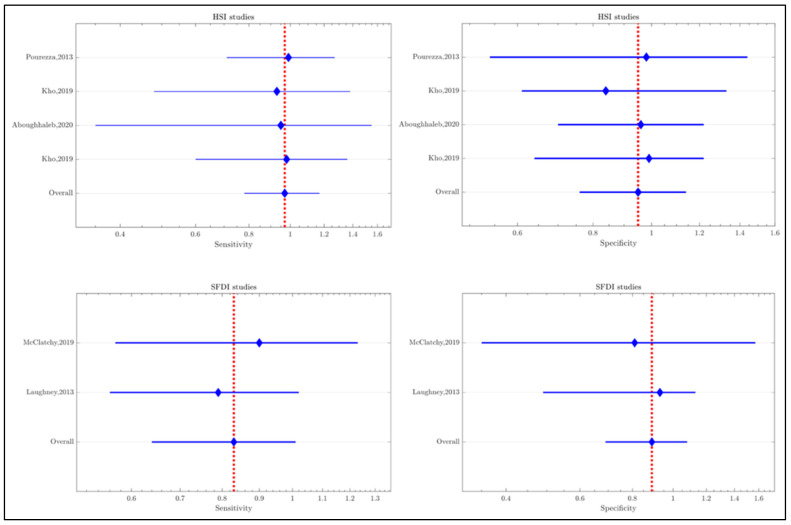
Forest plots for pooled sensitivity and specificity applied to the subdivisions within image based studies [37,38,42,43,44,45]. Forest plots for pooled sensitivity (**left**) and specificity (**right**) where a fixed model analysis was applied to the HSI studies [37,38,42,43] only (**top**) and to the SFDI studies [44,45] only (**bottom**).

The Forest plots depicting pooled sensitivity/specificity are presented in Figure 5 below for probe-based and imaging-based studies. The Forest plots depicting pooled sensitivity/specificity for the subdivisions within each modality can be found in Appendix A (Figure A1 and Figure A2). Finally, the results of using the Q-statistic to compare the pooled results between the two study types and subdivisions are presented in Table S3 (Supplementary Materials).

## 4. Discussion

### 4.1. Meta-Analysis of Probe-Based vs. Image-Based Approaches

According to the meta-analysis results presented in Table 8 and Figure 5, the probe-based technique’s pooled sensitivity/specificity (0.84/0.85) was inferior to that of the image-based method (0.90/0.92). However, when the Q-statistic was used to compare these pooled values, these differences were not statistically significant. There is insufficient evidence to support the hypothesis that probe-based modalities are inferior; however, there are several reasons why this meta-analysis presented these particular findings. First, the superiority of imaging could be attributed to the up-to-date and advanced image processing techniques used in these studies (e.g., U-Net, k-means clustering). Moreover, although the Q metric was very small for the image-based studies, it is unlikely that the between-study variance was negligible, as each of these studies employed different imaging instrumentation and image processing techniques. Table S4 in the Supplementary Materials highlights the variability in image processing techniques.

Another important consideration when it comes to diagnostic accuracy comparisons is a study’s statistical power. Although this information is not reported in the investigated studies, the quantity of spectral data acquired from imaging approaches is significantly higher than obtained from probe-based techniques. For example, Kho et al. gathered 224,861 spectra from 29 patients [39] with HSI. Similarly, SFDI produces a large volume of spectral data, with Laughey et al. gathering 265,000 pixels from 47 patients [39]. Comparatively, Keller et al. gathered 179 spectra from 40 patients.

Another limitation of comparing probe-based approaches to image-based approaches is that histological validation varied amongst the studies. Table S5 in the Supplementary Materials summarises the techniques used to correlate spectral readings to tissue ground truths. The main difference noted is that, with imaging, direct correlation with histology is more straightforward, as pathologists were able to annotate regions of interest. Notably, studies of probe-based approaches used a variety of methods to obtain histological ground truths.

### 4.2. Meta-Analysis of Modality Sub-Divisions

When comparing DRS studies against DRS with IFS studies, the sensitivity of the DRS approach (0.88 (95% CI: 0.82 to 0.95)—random model) was superior to the sensitivity of DRS combined with IFS (0.77 (95% CI: 0.67 to 0.87)—random model). This difference in sensitivities was not statistically significant (*p* = 0.07) according to the Q-statistic. The difference was less prominent and not statistically significant (*p* = 0.91) for the specificity of the two approaches: 0.87 (95% CI: 0.78 to 0.95– random model) for DRS and 0.86 (95% CI: 0.75 to 0.96– random model) for DRS with IFS. For imaging, HSI studies were observed to have higher sensitivity/specificity (0.97 (95% CI: 0.78 to 1.16)|0.95 (95% CI: 0.76 to 1.14) compared to the SFDI studies’ sensitivity/specificity (0.82 (95% CI: 0.64 to 1.01)|0.88(95% CI: 0.69 to 1.08)). However, the Q-statistic showed that these trends were not statistically significant (*p* = 0.28 for the sensitivity and *p* = 0.63 for the specificity comparisons).

### 4.3. Future Work

This review identified features that should be optimized in future optical IMA tools. The first question concerns the cancer-specific wavelengths that should be used in future systems. In the visible wavelength range, blood is a principal absorber of light. Therefore, intraoperatively, we should avoid measuring blood on the surface, although we have not identified any studies in breast surgery that have explored this effect. De Boer at al. studied the use of an extended NIRF range (1000–1600 nm), which reduces the effect of blood’s absorption of light [40]. Kho et al. found that the use of the visible spectrum and NIRF could better identify areas of DCIS [42] and that, in the NIRF range, water and fat are the main absorbers of light [52]. Their HSI system could discriminate between benign and malignant tissues at a depth of 2 mm. This spatial depth resolution could be deemed adequate, as the current clinical guidelines suggest that clear margins of 1 mm can reduce the local recurrence rates [4,53]. In comparison, Aboughaleb et al. [38] used only visible spectrum bands in their hyperspectral systems, achieving good discrimination between normal and malignant tissue. Future work must explore the ideal wavelength in the in vivo setting. Algorithms trained on ex vivo datasets may not be directly transferable to the in vivo setting, due to alterations in tissue physiology.

An ideal IMA tool provides the surgeon with rapid visualization and tissue characterization of the region of interest. Only six studies used whole field-of-view imaging modalities (Table 3). The rest all used probe-based techniques, with variable set-ups (Table 2). A limitation of probe-based techniques is that only a small area of tissue is sampled (around 1 mm^2^), which means the region of interest may be missed. It is difficult to survey a given resection margin of large surface area with reasonable resolution. Using a probe on multiple small areas of tissue can impose a time constraint in the surgical workflow. The use of a multi-channel device consisting of eight probes was used in three papers included in this review [12,24,29]. A multichannel device has the ability to scan an area up to 4.5 × 9.5 cm (40 cm^2^). This generates a spectral contour map. Brown et al. describe the strength of this multi-channel device as being able to focus on the sensitivity of tissue discrimination, rather than spatial resolution [12].

Speed is crucial to ensuring that any IMA tool used intraoperatively does not hinder the surgical workflow. This review determined that the time taken to gather data from a margin edge or specimen can range from seconds to minutes (Table 2 and Table 3). Aboughaleb et al. reported an HSI image capture time from 5 to 12 s, with a processing time of 20 s [38]. Similarly, Kho et al. developed an HSI system that takes 60 s to capture an image [42]. In comparison, SFDI systems take longer to image a specimen side, ranging from 5 to 10 min [54]. The evaluation methodologies of spectral readings are an important component of any optical system, and data processing techniques need to be optimized to allow for real-time feedback. Current spectroscopy-based classification procedures utilize signal processing methods, such as k-nearest neighbours classification and principal component analysis, either alone or combined with independent component analysis. The drawback of these algorithms is that they require a large database of cases with similar features to produce good reliability. Our review contains studies of small sample sizes. Breast cancer is highly heterogenous and any future work needs to include high patient numbers to account for tumour and patient variability, in order to train datasets accordingly.

Breast tissue is heterogenous, and further heterogeneity arises when one takes into account whether a patient is pre- or post-menopausal [55]. Pre-menopausal women have more fibroglandular tissue and denser breast tissue. Brown et al. aimed to account for this interpatient variation. They discovered that patients with higher mammographic breast density were associated with higher baseline B-carotene concentrations and higher scattering coefficients [14]. Boer et al. determined that using the fat/water ratio is a good discriminator between benign and malignant tissues. They recommend that, intraoperatively, the surgeon should use the probe at benign spots to set a reference level. Not all studies evaluated in this review offer a clear description of patient demographics, and we recommend future studies take this into consideration.

To ensure that a tissue optics method is accurate in discriminating normal and malignant breast tissues, the results must be correlated using histopathology. A weakness of the meta-analysis conducted in this review is that the studies used different methods to correlate optical spectral data with histopathology. A common challenge in this field is to develop robust classification algorithms, as there is often a spatial mismatch between optical measurements and histopathology. For instance, during tissue fixation by the pathologist, there is tissue shrinkage; therefore, the spatial correlations between the specimen and the stained slides differ.

## 5. Conclusions

Future work on IMA tools must take into consideration several factors to create a rapid, non-contact device that confers accuracy in discriminating between normal and malignant breast tissues. First, the wavelengths of light to be used in any device must be selected carefully. Spatial resolution and depth resolution are crucial, as identifying small regions of DCIS, for example, is what makes an IMA tool useful in preventing the need for re-excision surgery. Speed and the data processing time are crucial to a surgical workflow pattern.

## Figures and Tables

**Table 1:** Summary of the modality and wavelength range for the 19 included studies.

Author	Year	Modality Type	Probe or Imaging Based	Wavelengths Used (nm)
Nachabe [39]	2011	DRS	Probe	500–1600
de Boer [40]	2015	DRS	Probe	400–1600
Zhu [29]	2006	DRS	Probe	350–600
Brown [14]	2013	DRS	Probe	450–600
Evers [41]	2013	DRS	Probe	400–1700
Brown [30]	2010	DRS	Probe	381–630
Zhu [31]	2005	DRS-IFS	Probe	300–440
Volynskaya [32]	2008	DRS-IFS	Probe	300–800
Breslin [33]	2004	DRS-IFS	Probe	300–600
Keller [46]	2007	DRS-IFS	Probe	400–850
Palmer [34]	2003	DRS-IFS	Probe	300–600
Ramanujam [35]	2009	DRS-IFS	Probe	380–780
Keller [36]	2010	DRS-IFS	Probe	300–600
Pourezza-Shahri [37]	2013	HSI	Imaging	380–780
Kho [43]	2019	HSI	Imaging	953–1645
Aboughhaleb [38]	2020	HSI	Imaging	420–620
Kho [42]	2019	HSI	Imaging	450–1650
McClatchy [44]	2019	SFDI	Imaging	658,730,850
Laughney [45]	2013	SFDI	Imaging	658,730,850,970

**Table 2:** Characteristics of the probe-based systems of studies included in this review. The parameters evaluated include probe geometry, acquisition time, area covered, and probe depth. Not all papers documented such parameters.

Author	Probe Type	No. of Fibres	Distance between Fibres	Acquisition Time	Sensing Area	Probing Depth
Nachabe [39]	Single (1.3 mm)	X3 200 μm core diameter fibres; x1 connected to light source	2.48 mm	0.5 s	-	-
de Boer [40]	Single (1.75 mm)	-	1.5 mm	20 min for 55 grid points (2.75 min)	Probing volume = 1–3 mm^3^	-
Zhu [29]	Single	Illumination core (19 fibres)	-	0.025 s/spectra	-	0.5–2 mm
Brown [14]	Multichannel (8 channels)	-	10 mm between each channel	10 min per margin (8 spectra acquired per probe placement)	-	0.5–2.2 mm
Evers [41]	Single (1.3 mm)	X3 core fibres (x1 light; x1 NIRF and x1 visual spectrometer)	2.48 mm	0.2 s	5 mm^2^	
Brown [30]	Multichannel (8 channels)	8 channels (19 illumination fibres; 4 collection fibres)	10 mm between each channel	40 s	1.5 cm × 5.5 cm	0.5–2.2 mm
Zhu [31]	Single	Illumination core (19 fibres); x3 collection rings (12 fibres)	3 illumination–collection separations 735/980/1225 μm	1 min (for 8 fluorescence spectra and 1 DRS spectra)	-	-
Volynskaya [32]	Single	1 delivery fibre; x6 collection fibres	-	1.5 s	-	100 μm
Breslin [33]	Single	Central collection region; outer ring excitation fibres	-	-	-	-
Keller [46]	Single	x7 fibres—300 μm, in a six-around-one configuration	-	100 ms/spectra; 60 s per margin	25 mm × 25 mm	-
Palmer [34]	Single	31 fibres (central collection core diameter 1.52 mm; illumination ring outer diameter 2.18 mm)	-	8 min	-	1050 μm
Ramanujam [35]	Multichannel (8 channels) with 19 illumination and 4 collection fibres in each	Illumination core (19 fibres—200 μm); x4 collection fibres (200 μm)	-	-	-	-
Keller [36]	Single	Core (7 fibres—300 μm)	-	60–90 s per margin	-	-

**Table 3:** Parameters extracted from the imaging studies included spatial resolution, area of field-of-view (FOV) the imaging system can capture, the time taken to conduct the imaging, and depth penetration. Not all studies discussed these parameters.

Author	Modality	Type of HSI	Spatial Resolution	FOV	Time
Pourezza-Shahri [37]	HSI	Wavelength filtering	150 microns per pixel-	−768 × 1024 pixels	1 min
Kho [43]	HSI	Pushbroom	Each pixel equates to 0.5 mm.	200 lines scanned per patient (each line = 320 pixels)	4 s
Aboughaleb [38]	HSI	Pushbroom	Each pixel was 0.22 mm × 0.22 mm	-	Capture time 5–12 s; Processing time 20 s
Kho [42]	HSI	Pushbroom	0.16 and 0.5 nm/pixel	12.5 × 18 cm	20 s for NIR; 40 s for VIS
McClatchy [44]	SFDI	-	-	-	-
Laughney [45]	SFDI	-	30 spatial frequencies distributed between 0 and 0.33 mm^−1^	5.5 inch × 7.5 inch	10 min, 360 images per specimen

**Table 4:** Patient ages and tissue types utilized in the studies assessed in this review. In total, 15 studies characterized tissue samples into malignant and non-malignant types. Only 10 studies subdivided tissue types into various histological subtypes. (FA—fibroadenoma; IDC—invasive ductal carcinoma; ILD—invasive lobular carcinoma; DCIS—ductal carcinoma in-situ). Only three studies mentioned neither patient ages nor tissue types [30,35,38].

Author	Mean Age	No. of Malignant Samples	No. of Non-Malignant Samples	Adipose	Glandular	FA/Fibrous	IDC	ILC	DCIS
Nachabe [39]	-	29	73	43	23	7	21	0	8
de Boer [40]	-	25 (102 from tumour border)	42	-	-	-	-	-	-
Zhu [29]	-	35	50	39	1	10	28	1	2
Brown [14]	-	46	42	-	-	-	14	-	17
Evers [41]	52	59	148	79	37	32	30	5	24
Zhu [31]	-	13	34	20	2	12	7	4	2
Volynskaya [32]	-	9	95	31 samples were normal		64	9 samples were invasive		-
Breslin [33]	48.4 (51.5 for cancer)	20	36	21	15 samples were glandular/fibrous		16	2	1
Keller [46]	-	27	102	-	-	-	-	-	-
Palmer [34]	-	20	36	21	15 samples were glandular/fibrous		16	2	1
Keller [36]	-	34	145	-	-	-	-	-	-
Pourezza-Shahri [37]	-	14	33	-	-	-	-	-	-
Kho [43]	67 ± 1	-	-	-	-	-	-	-	-
Kho [42]	57 ± 11	13	18	13	5	-	10	-	3
McClatchy [44]	-	10	21	5	16 samples were fibroglandular		8	2	-
Laughney [45]	-	27	20	-	9	11	24	1	2

**Table 5:** The number of breast tissue samples or patients in each study is recorded. Certain studies document how many spectral measurements were recorded. The sensitivity and specificity of the ability to distinguish between normal and invasive malignant tissues is tabulated as below. Notably, some studies also recorded the sensitivity/specificity of the ability to identify DCIS; however, these figures were not used in the pooled statistics.

Author	Modality Type	No. of Samples/Locations	No. of Spectral Measurements	No. of Lumpectomies	No. of Patients	Sensitivity (%)	Specificity (%)
Nachabe [39]	DRS	102	980	-	52	91	95
de Boer [40]	DRS	169	169	16	-	100	100
Zhu [29]	DRS	85	-	-	45	83.9	88.6
Brown [14]	DRS	88	-	-	70	74	86
Evers [41]	DRS	207	1073	-	47	90	88
Brown [30]	DRS	56	-	-	48	79	66.7
Zhu [31]	DRS-IFS	47	-	-	18	61.54	82.35
Volynskaya [32]	DRS-IFS	104	202	-	17	100	96
Breslin [33]	DRS-IFS	56	-	-	32	70	71.1
Keller [46]	DRS-IFS	129	129	-	24	78	99
Palmer [34]	DRS-IFS	56	-	-	32	70	92
Ramanujam [35]	DRS-IFS	55	-	-	48	79	67
Keller [36]	DRS-IFS	-	179	-	40	85	96
Pourezza-Shahri [37]	HSI	47	-	19	-	99	98
Kho [43]	HSI	18	22,000	6	18	93	84
Aboughhaleb [38]	HSI	10	-	10	-	95	96
Kho [42]	HSI	26	24,539	-	42	98	99
McClatchy [44]	SFDI	31	50,521	31	29	90	81
Laughney [45]	SFDI	59	265,000	-	47	79	93

**Table 6:** Heterogeneity of probe-based studies versus imag- based studies. The posterior probability for heterogeneity was calculated for a prior probability for heterogeneity of Pr(Het) = 0.5.

**Sensitivity Analysis**				
	Q-statistic	*p*-value	Q-test power	Pr(Het|Q)
Probe-based studies	17.48	0.09	~10^−5^	0.51
Image-based studies	1.5	0.91	0.02	0.51
**Specificity Analysis**				
	Q-statistic	*p*-value	Q-test power	Pr(Het|Q)
Probe-based studies	16.27	0.18	~10^−5^	0.51
Image-based studies	0.92	0.97	0.02	0.51

**Table 7:** Heterogeneity based on modality subdivisions. The posterior probability for heterogeneity was calculated for a prior probability for heterogenety of Pr(Het) = 0.5.

**Sensitivity Analysis**				
	Q-statistic	*p*-value	Q-test power	Pr(Het|Q)
DRS	5.7	0.34	0.03	0.51
DRS with IFS	8.13	0.15	0.07	0.50
HSI	0.06	1	0.08	0.49
SFDI	0.29	0.59	0 (1 study)	0.51
**Specificity Analysis**				
	Q-statistic	*p*-value	Q-test power	Pr(Het|Q)
DRS	7.2	0.21	0.05	0.50
DRS with IFS	8.29	0.14	0.67	0.26
HSI	0.35	0.95	0.08	0.49
SFDI	0.34	0.56	0 (1 study)	0.51

**Table 8:** Pooled sensitivity/specificity results for probe-based vs. image-based approaches—fixed (random). Results indicated with an asterisk (*) are categories where the Q-statistic is very small (Q < 1).

	**Sensitivity analysis**		
	Pooled	Lower limit	Higher limit
Probe-based studies	0.84 (0.83)	0.78 (0.76)	0.89 (0.90)
Image-based studies	0.90 (0.91)	0.76 (0.82)	1.03 (1)
	**Specificity analysis**		
	Pooled	Lower limit	Higher limit
Probe-based studies	0.85 (0.85)	0.79 (0.78)	0.91 (0.92)
Image-based studies	0.92 (0.92) *	0.78 (0.78) *	1.06 (1.06) *

## Data Availability

Data is contained within this article, and the Appendix A and Supplementary Materials.

## Supplementary Materials

The following supporting information can be downloaded at https://www.mdpi.com/article/10.3390/cancers15112884/s1: Supplementary Materials S1: Search strategy; Table S1: Pooled sensitivity/specificity results based on modality subdivisions; Table S2: Comparison of pooled sensitivity/specificity results with the use of the Q-statistic; Table S3: Processing techniques used in each study for spectral data analysis; Table S4: Ground truth methods used for the histological validation of the spectral readings.
