# Supplementary material for: A Comparison of Spectroscopy and Imaging Techniques Utilizing Spectrally Resolved Diffusely Reflected Light for Intraoperative Margin Assessment in Breast-Conserving Surgery: A Systematic Review and Meta-Analysis
Source: Cancers (Basel). 2023 May 23;15(11):2884. doi: 10.3390/cancers15112884 (PMC10251883; doi:10.3390/cancers15112884)
Supplement: Supplementary file 1 [file cancers-15-02884-s001.zip › cancers-2370065-supplementary.pdf]

Supplementary Materials

Supplementary S1. Search Strategy used for Systematic Review. Medline, Embase and Scopus databases were used.

SCOPUS
"diffuse optical spectroscopy imaging"
"diffuse reflection spectroscopy"
"diffuse reflectance spectroscopy"
"diffuse optical spectroscopic imaging"
"multi-spectral imaging"
"multispectral imaging"
"hyperspectral imaging"
"hyper-spectral imaging"
"optical imaging"
"spatial frequency domain imaging"
multispectral W/2 imaging
hyperspectral W/2 imaging
hyperspectral W/2 imag*
multispectral W/2 imag*
AND
"breast cancer"
breast tumo?r
"breast carcinoma"
"breast neoplasm"
breast W/2 cancer
"lumpectomy"
breast AND lesion*
"breast W/2 surg*"
"breast W/1 operation

MEDLINE
Breast cancer.mp or exp Breast Neoplasms/
Breast tumo?r.mp
Breast adj2 carcinoma.mp
Breast lesion*.mp
Lumpectomy.mp or exp Mastectomy, segmental/
Breast adj2 surgery
Breast adj2 operation
AND
Optical imaging.mp or exp Optical Imaging/
Multispectral adj2 imaging
Hyperspectral adj3 imag*
Exp Hyperspectral Imaging/
Diffuse optical spectroscopy imaging.mp
Diffuse optical spectroscopic imaging.mp
Diffuse reflectance spectroscopy.mp
Diffuse reflection spectroscopy.mp
Spatial frequency domain imaging.mp

EMBASE
Breast cancer.mp or exp breast cancer/
Exp breast tumor/ or breast neoplasm*.mp
Breast tumo?r.mp
Breast adj2 carcinoma.mp
Exp breast lesion/ or breast lesion*.mp
Lumpectomy.mp or exp lumpectomy/

Breast adj2 surgery.mp
Breast adj2 operation.mp
AND
Optical imaging.mp
Multispectral adj2 imag*
Hyperspectral adj3 imag*
Diffuse optical spectroscopy imaging.mp
Diffuse optical spectroscopic imaging.mp
Diffuse reflectance spectroscopy.mp or exp diffuse reflectance spectroscopy/
Diffuse reflection spectroscopy.mp
Spatial frequency domain imaging.mp

Table S1. Pooled sensitivity/specificity results based on modality subdivisions – fixed (random)

	**Sensitivity analysis**		
	Pooled	***Lower limit***	***Higher limit***
DRS	0.88 (0.88)	0.81 (0.82)	0.95 (0.95)
DRS with IFS	0.78 (0.77)	0.70 (0.67)	0.86 (0.87)
HSI	0.97 (0.97)*	0.78 (0.78)*	1.16 (1.16)*
SFDI	0.82 (0.82)*	0.64 (0.64)*	1.01 (1.01)*
	**Specificity analysis**		
	Pooled	***Lower limit***	***Higher limit***
DRS	0.86 (0.87)	0.78 (0.78)	0.94 (0.95)
DRS with IFS	0.86 (0.86)	0.78 (0.75)	0.95 (0.96)
HSI	0.95 (0.95)*	0.76 (0.76)*	1.14 (1.14)*
SFDI	0.88 (0.88)*	0.69 (0.69)*	1.08 (1.08)*

Table S2. Comparison of pooled sensitivity/specificity results with the use of the Q-statistic. P-values are less than 0.05 showing that the differences are not statistically significant at 5% significance level.

**Pooled Sensitivity Comparisons**		
**Studies being compared**	***Q statistic (p-value) fixed-model***	***Q statistic (p-value) random-model***
Probe-based vs image-based	0.64 (0.42)	1.85 (0.17)
DRS vs DRS with IFS	3.66 (0.06)	3.22 (0.07)
HSI vs SFDI	1.16(0.28)	1.16 (0.28)
**Pooled Specificity Comparisons**		
**Studies being compared**	***Q statistic (p-value) fixed-model***	***Q statistic (p-value) random-model***
Probe-based vs image-based	0.86 (0.35)	0.75 (0.39)
DRS vs DRS with IFS	10^{-6} (~1)	0.01 (0.91)
HSI vs SFDI	0.24(0.63)	0.24(0.63)

Table S3. Processing techniques used in each study for spectral data analysis.

Nachabe[39]	Diffusion theory model to extract quantitative clinical parameters.	The classification and regression tree (CART) algorithm.

Study	*Feature extraction method used*	*Classification Method*
de Boer[40]	Diffusion theory model to extract quantitative clinical & physical parameters.	Fat to water content ratio (F/W) value threshold: F/W=1.
Zhu[29]	Inverse Monte Carlo model to extract quantitative optical parameters or partial least squares (PLS) to extract principal components in the measured DRS.	Wilcoxon rank-sum test for feature selection and support vector machine (SVM) for classification.
Brown[14]	Inverse Monte Carlo model to extract 5 quantitative physical parameters and 3 image descriptive variables (optimum threshold, median, Kolmogorov-Smirnov statistic) for each of the extracted physical parameters.	Conditional inference tree (CIT) model for feature selection and classification.
Evers[41]	Diffusion theory model	CART algorithm
Brown[30]	Inverse Monte Carlo model to extract β-carotene and total haemoglobin values.	Receiver Operator Characteristic (ROC) analysis & Youden index to identify β-carotene and total haemoglobin threshold values. Decision tree-based two parameter model for classification.
Zhu[31]	Principal Component Analysis (PCA) on the DRS and IFS spectra for principal components.	Wilcoxon rank-sum test for principal components selection and support vector machine (SVM) for classification.
Volynskaya[32]	Diffusion theory model to extract scattering, oxyhaemoglobin, β-carotene parameters. IFS with multivariate curve resolution (MCR) to extract NADH and collagen-like parameters.	3-step binary classification algorithm using logistic regression.
Breslin[33]	Principal component analysis on the DRS and IFS spectra for principal components.	Wilcoxon rank-sum test for principal components selection and support vector machine (SVM) for classification
Keller[46]	Maximum representation and discrimination feature (MRDF)	Sparse multinomial logistic regression (SMLR)
Palmer[34]	Principal component analysis on the DRS and IFS spectra for principal components.	Wilcoxon rank-sum test for principal components selection and support vector machine (SVM) for classification
Ramanujam[35]	Inverse Monte Carlo model to extract β-carotene and total haemoglobin values.	Wilcoxon rank-sum test for parameter selection. Decision tree-based two parameter model for classification.

Keller[36]	Maximum representation and discrimination feature (MRDF) to extract the diagnostically relevant DRS and IFS features.	Sparse multinomial logistic regression (SMLR)
Pourezza-Shahri[37]	Fourier coefficient s election features (FCS) for feature extraction.	Minimum Redundancy Maximum Relevance (MRMR) method for feature selection. SVM with Radial Basis Function (RBF) kernel for classification.
Kho[43]	Processed spectral band values were used as features.	Linear, weighted support vector machine (SVM).
Aboughhaleb [38]	-	Contour Mapping with the K-means image clustering up to 8-clusters with variable threshold value from 0.2 up to 0.9 and statistical analysis to identify best threshold.
Kho[42]	1) Fisher's linear discriminant analysis (LDA) for feature extraction.	1) Pairwise coupling for classification for the LDA-derived features.<br>2) U-Net for image segmentation/ classification based on spatial-spectral image features.
McClatchy [44]	Minimization of sum of squares residual error between measured and diffusion theory model simulated reflectance to extract optical properties.	Fitted curves between histological fractions and each scattering parameter for optical properties selection. Use of optical properties to extract adipose volume fraction and epithelium to stroma ratio values for classification.
Laughney [45]	Minimization of sum of squares residual error between measured and Monte Carlo simulated modulation amplitude to extract optical parameters.	K-dimensional decision tree-based model for classification of a parameter set based on the known classification of the k nearest neighbours (from training set).

Table S4. Ground truth methods used for histological validation of spectral readings.

Author	Ground Truth
Nachabe[39]	5 tissue types (adipose;glandular; fibroadenoma; invasive cancer; DCIS) macroscopically identified by a p measurements taken, and co-registered with pathology findings.
de Boer[40]	Lumpectomy specimens inked, cooled in a freezer and sliced. 3 classes of measurements taken tumour/border/middle of tumour). Following H&E staining, multiple sections of each lumpectomy slic probe at each measurement location. Correlation of macroscopic and microscopic images.
Zhu[29]	Probe placed on whole lumpectomy specimen. Area inked where probe placed. Transverse sections cut stained as per H&E protocol. The thickness of each sample measured from the inked spot to the bottom
Brown[14]	DRS measurements collected from tissue surface, until whole margin had beeen measured.<br>a) Margin Level Histology – 4 corners of measured margin marked with ink to allow for patholog<br>b) Site Level H&E Image Analysis – 10 sites per margin were inked
Evers[41]	Spectra collected from macroscopically selected areas (normal; fat; glandular tissue; fibroadenoma lesion

	Following H&E protocol, a blinded pathologist read slides. The % of different tissue types at each locatio[illegible] at each location should contain at least 20% of each type.
Brown[30]	DRS measurements collected from tissue surface, until whole margin had beeen measured. a) Margin Level Histology – 4 corners of measured margin marked with ink to allow for patholog[illegible] b) Site Level H&E Image Analysis – 10 sites per margin were inked
Zhu[31]	Probe placed on whole lumpectomy specimen. Area inked where probe placed. Transverse sections cu[illegible] stained as per H&E protocol. The thickness of each sample measured from the inked spot to the bottom [illegible]
Volynskaya[32]	Spectral data collected from multiple sites identified by the pathologist. Probe spot marked with ink.
Breslin[33]	Pathologist gross evaluation of samples tumour/adjacent normal appearing tissue from lumpectomy sp[illegible] to be more than 1cm from grossly visible tumour margin to minimize the potential of measuring DCIS. A[illegible] spot was inked. Following H&E protocol microscopic evaluation of each location was made.
Keller[46]	Spectral data gathered from one point on each face of the specimen. Probe locations marked with ink so ab[illegible]
Palmer[34]	Follow surgical resection, samples of normal and tumour tissue are cut from a larger specimen. Norma[illegible] from tumour. Probe point inked. Microscopic evaluation of each section performed.
Ramanujam[35]	Margin level pathology assessed. 4 corners of the imaged margin is marked with ink to establish the b[illegible] pathology report is used to correlate spectral findings with.
Keller[36]	Spectral data is acquired from each side of a specimen (6 sides in total). Probe location marked with ink. [illegible] at marked locations to enable correlation with spectral data.
Pourezza-Shahri[37]	A pathologist tags several regions of an image for histological analysis. Optical data taken from region [illegible] are fed into image classification software. Every pixel within a region of interest is categorised into eithe[illegible]
Kho[43]	Tissue slices – following a lumpectomy, the specimen was sliced. Hyperspectral measurements were [illegible] staining of each slice, annotations were made idenitifying different classes within each slice. Then pix[illegible] labelled as invasive cancer; DCIS; adipose or connective tissue. Resection surface – each side of a lumpectomy specimen (6 in total) were imaged. Following inking of ea[illegible] the nipple to the periphery. 66 slices created, but only 43 treatd via H&E. The authors note that the perpe[illegible] to the resection surface differ and therefore it is not possible to verify the entire resection surface. Th[illegible] invasive cancer/DCIS was within 2 mm of a resection surface.
Aboughhaleb[38]	Breast tissue cut into 3cm x 2cm samples, and kept in an ice box, stored at -70 degrees. Samples brought [illegible] imaged. Healthy tissue was regarded to be 5 – 10 cm away from tumour tissue, amd was confirmed by [illegible] do not mention further pathological involvement in the paper.
Kho[42]	A fresh breast specimen was sliced and HSI images taken, and then processed via H&E staining. Di[illegible] correlated with HSI images. Tissue fixation can cause shrinkage and this was corrected for. 4 tissue class[illegible] DCIS; connective tissue; adipose). The authors note that the diffuse reflectance spectrum of a specific pix[illegible] type located 1mm distance away. They calculated datasets for 'all histopathology labels' as well as 'relia[illegible]
McClatchy[44]	Tissue grossly identified as either invasive cancer; fibroglandular; fibroadenoma or adipose tissue. The [illegible] 25mm x 5 cm chunks to be imaged. Following H&E protocol, the slides were digitalized. The fraction o[illegible] each lesion was calculated. Regions of interest were mapped out, and the authors identify and commen[illegible] from optical property maps due to fixation.

Laughney[45]	A fresh specimen is sliced. Tissue slices are imaged. The exact area of tissue imaged is outlined with 4 ink block. Following formalin fixation (with pins in situ), histological analysis allowed representation of imag tissue areas containing breast lesions.
